# The relative citation ratio: what is it and why should medical librarians care?

**DOI:** 10.5195/jmla.2018.499

**Published:** 2018-10-01

**Authors:** Alisa Surkis, Stuart Spore

**Affiliations:** Assistant Director for Research Data and Metrics, Health Sciences Library, NYU, 577 1st Avenue, New York, NY 10016; Lead, Scholarly Output Assessment, Health Sciences Library, NYU, 577 1st Avenue, New York, NY 10016

## Abstract

Bibliometrics is becoming increasingly prominent in the world of medical libraries. The number of presentations related to research impact at the Medical Library Association (MLA) annual meeting has been increasing in past years. Medical centers have been using institutional dashboards to track clinical performance for over a decade, and more recently, these institutional dashboards have included measures of academic performance. This commentary reviews current practices and considers the role for a newer metric, the relative citation ratio.

To judge by the program for MLA ’17, the 2017 Medical Library Association (MLA) annual meeting, bibliometrics is becoming increasingly prominent in the world of medical libraries. The number of presentations related to research impact at the MLA meeting has been increasing in past years, with a more than threefold increase between 2013 and 2017 (supplemental Appendix A). Medical centers have been using institutional dashboards to track clinical performance for over a decade [1], and more recently these institutional dashboards have included measures of academic performance [2]. Clearly, demand for metrics of all types is on the rise. Given this growing interest in and use of metrics, it is a good time to review current practices and consider the role for a newer metric.

Most medical librarians are familiar with the journal impact factor (JIF). While this metric provides value in specific use cases, it is often used inappropriately. The JIF was devised to measure the influence of journals but has been widely used to judge individual articles. This use is based on the specious assumption that all articles in a journal are equally influential, as reflected by their number of citations [3, 4]. This misuse can lead to weak articles being overvalued and important work being undervalued.

The h-index [5] is another familiar metric. It can be problematic as well, being in essence the product of a simple, somewhat arbitrary, formula, which derives a single number from a list of citation counts. While often used to compare researchers, the h-index can be misleading when comparing researchers in different fields because it does not take into account the differing citation practices of different fields [6]. It also disadvantages newer investigators, as the value of the h-index is driven by the number of articles authored, regardless of how many citations those articles may attract [7].

The relative citation ratio (RCR) is a prominent new metric that is more appropriate for many common-use cases. The RCR was developed by the National Institutes of Health (NIH) Office of Portfolio Analysis as part of an effort to improve evaluation of grant outcomes and was presented as a preprint in 2015 [8] and then as a peer-reviewed article in *PLOS Biology* in 2016 [9]. In contrast to journal-level metrics like the JIF and author-level metrics like the h-index, the RCR is an article-level metric. While there are a number of article-level citation metrics, the RCR has several properties that make it a particularly appealing choice.

The RCR uses a novel approach to normalize the metric for the “discipline” of the article, with the idea that the metric can then be compared between articles in different fields. This technique is known as field normalization. While there are other citation-based, article-level metrics that employ some type of field normalization, previous metrics have mainly done so by assigning an article to a field or set of fields chosen from a fixed set of categories [10–13]. The RCR uses an approach that dynamically determines the field of the article based on the co-citation network of that article, that is, all articles that have been cited by articles citing the target article [9]. As science becomes increasingly interdisciplinary, this approach takes into account that the field of an article may not be well captured by a predefined discipline or set of disciplines and that, as disciplines evolve, the discipline that best captures an article may also evolve.

While somewhat complicated, the RCR is transparent (particularly when compared with proprietary metrics). The details of its calculation are clearly spelled out in the Hutchins article [9]. NIH provides access to the RCR of articles through a web interface [14] and an application programming interface (API) [15]. Other popular field-normalized, article-level metrics are proprietary, with less transparency in their calculation, and are available only through licensing agreements.

Finally, a key aspect of the derivation of the RCR is that it is benchmarked to NIH R01-funded articles. The calculation of this metric is done such that an article with an RCR value equal to 1.0 is at the median for NIH R01-funded articles for that year. This leads to a clear interpretation of RCR values, in that an RCR value of 1.0 is the demarcation between articles that have a lower citation rate than the median NIH R01-funded publication and those with a higher citation rate. Because the RCR makes it straightforward to communicate how a particular article compares to NIH R01-funded works, it has the potential to be a particularly compelling metric among users focused on NIH funding as a primary measure of impact.

The following examples illustrate some of the pitfalls in assessing research impact that the RCR can help avoid. The first example compares two articles published in 2005:

1. Mallucci L, Wells V. Potential role of the antiproliferative cytokine beta-galactoside binding protein in cancer therapy. Curr Opin Investig Drugs. 2005 Dec;6(12):1228–33. PMID: 16370387.
2. Ogedegbe G, Cassells AN, Robinson CM, DuHamel K, Tobin JN, Sox CH, Dietrich AJ. Perceptions of barriers and facilitators of cancer early detection among low-income minority women in community health centers. J Natl Med Assoc. 2005 Feb;97(2):162–70. PMID: 15712779.

The first article appears in a journal with a high JIF (15.333), whereas the second article appears in a journal with a very low JIF (0.182). Conversely, the first article has an RCR of 0.2, significantly below the NIH R01-funded median, whereas the second article has an RCR of 4.0, far above that median. This example illustrates how misleading reliance on the JIF for article-level comparisons can be.

Next, consider two articles published in 2013:

1. Djiane A, Krejci A, Bernard F, Fexova S, Millen K, Bray SJ. Dissecting the mechanisms of Notch induced hyperplasia. EMBO J. 2013 Jan 9;32(1):60–71. DOI: http://dx.doi.org/10.1038/emboj.2012.326. Epub 2012 Dec 11. PubMed PMID: 23232763.
2. Browning DJ. Impact of the revised American Academy of Ophthalmology guidelines regarding hydroxychloroquine screening on actual practice. Am J Ophthalmol. 2013 Mar;155(3):418–28.e1. DOI: http://dx.doi.org/10.1016/j.ajo.2012.09.025. Epub 2012 Dec 4. PubMed PMID: 23218706.

Both articles have 27 citations; however, the first article is in the field of genomics where articles tend to be much more heavily cited than in ophthalmology, the field of the second article. The RCR reflects this, as the genomics article has an RCR of 1.3, while the ophthalmology article with the same number of citations has an RCR of 3.7. A comparison of raw citations—or any metric based solely on those raw citation numbers—would have led to a very misleading comparison of the impact of these articles in their respective fields.

A recent example from our institution provides another striking illustration of the difference between assessing an article with the JIF versus the RCR. The New York University (NYU) Office of Science and Research requested a report on 2016 articles by NYU School of Medicine faculty from the database of faculty articles maintained by the library [16], including a section on articles appearing in journals with a JIF greater than 10. Upon discussion, it became clear that the office intended to identify works of particular merit. In response, the authors provided the requested report, along with an additional report listing works with an RCR in the 90th percentile or above relative to NIH R01-funded works. The reports listed 475 articles in journals with a JIF greater than 10 and 666 with RCR values at the 90th percentile or above (supplemental Appendix B), with only 186 of those articles in appearing in both lists.

Comparing the 100 highest-ranking items from both reports, we found only 36 articles common to the RCR list and JIF list. While the lowest impact factor in the top 100 list for JIF was over 30, the RCR top 100 list contained 36 articles from journals with a JIF less than 10. The JIF top 100 list contained 12 items with an RCR of less than 1.0 or no calculated RCR at all (due to inadequate numbers of recorded citations). To summarize, the 2 lists agreed on just over a third of their contents. Of the articles that the JIF flagged as most important, 12% showed no evidence of importance according to the RCR. Almost 40% of the articles that the RCR flagged as most impactful were published in journals with a JIF of less than 10.

To further explore the relationship between the 2 metrics, we calculated the correlation between the RCR and the JIF for research articles published by NYU School of Medicine faculty in 2015. We found 4,470 research articles (supplement Appendix C) and filtered out articles with either no JIF or no RCR. We performed linear regression of the RCR on the JIF for the resulting 3,772 articles, and the value of R-squared as 0.13 (this metric ranges between 0 and 1.0 and reflects the percent of variance in the RCR that is explained by the JIF). So, while the JIF and RCR are correlated with a high degree of certainty (*p*<2×10–16), the strength of that correlation is not particularly strong (Figure 1). Because only 13% of the variance of the RCR is explained by the JIF, the data points are not tightly clustered around the regression line, suggesting that the JIF serves as a very poor proxy for the impact of an article.

**Figure 1:**
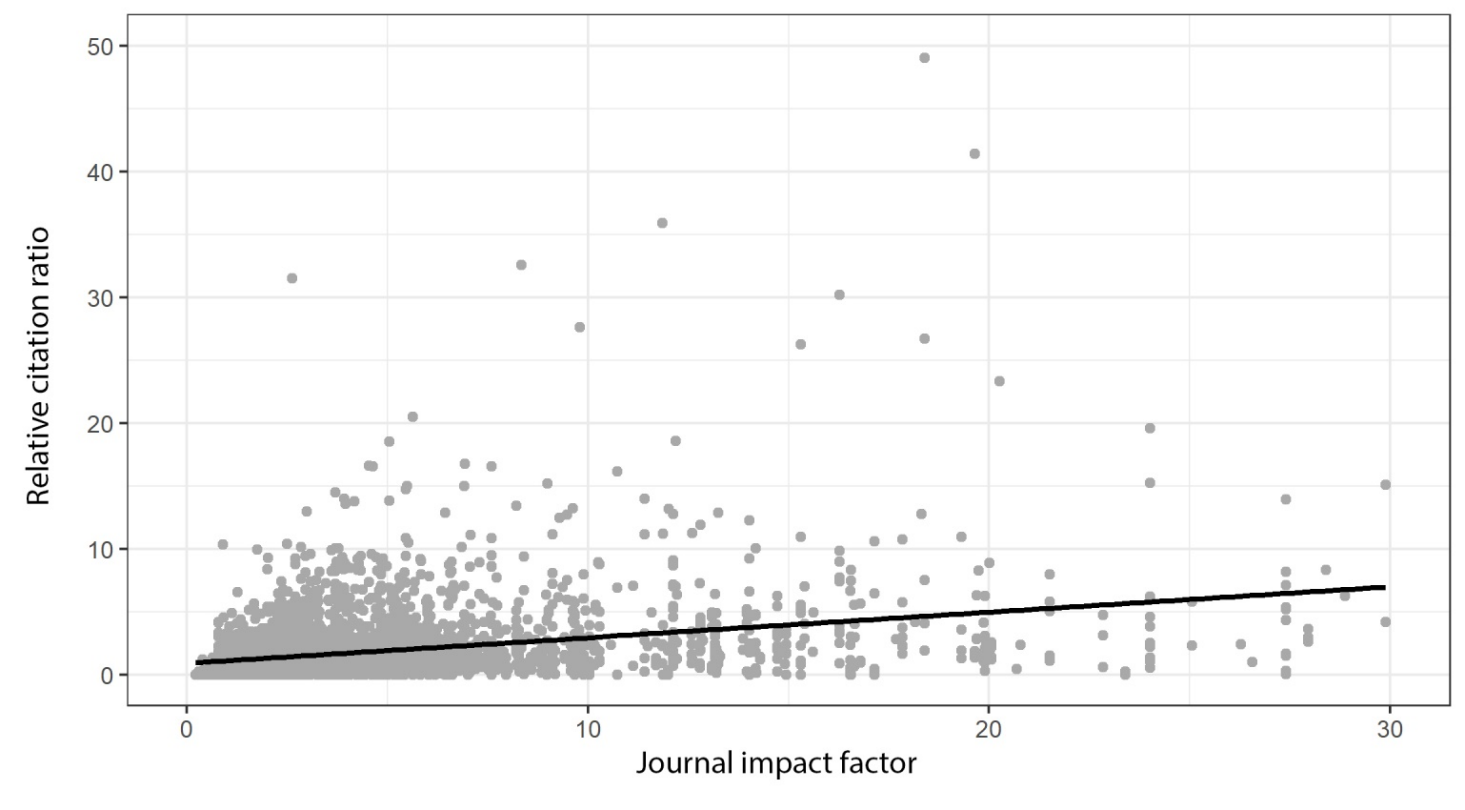
Relative citation ratio (RCR) versus journal impact factor (JIF) for research articles published in 2015 by NYU School of Medicine faculty, for which both an RCR and a JIF are available Display truncated to improve readability, resulting in 77 of the 3,772 points used in the regression not appearing due to high values of either RCR or JIF.

The above examples provide evidence that using the JIF as a proxy for the impact of an article can be very misleading. This has implications for the culture of faculty publishing. Over the last few decades, faculty who wanted their works to be recognized as important by hiring committees and promotion and tenure committees typically needed to ensure that they appeared in journals with high JIFs. By changing the focus from the JIF to the RCR, the focus shifts from “where” to “what”; thus, making journal choice alone less of a priority.

The RCR is just beginning to acquire “mind share” amongst practitioners, but bibliometricians have been discussing it intensely [4, 17–21], and there are active projects using the RCR for real-world data analysis [22–27]. Predictably, the RCR has not escaped criticism. As with all metrics, it has limitations and complications. The RCR is currently only calculated for MEDLINE citations from 1995 on, so it is not a suitable choice for older or for non-biomedical literature. Like other bibliometric measures, the RCR is subject to a good deal of latency (it takes time for citation counts to become meaningful), and therefore, like all citation-based metrics, it is of limited utility for newly published articles.

Another limitation is that, because the RCR uses NIH funding as its benchmark, its usefulness might be diminished outside of the United States. It has also been suggested that the RCR’s strength in field normalization might result in it discounting the value of interdisciplinary works [21]. Finally, it is possible for the addition of new citations to cause a decrease in the RCR [18], either because the field of the article changes over time or because the article accrues citations at a slower pace than other articles published in that year. However, analyses show that even a small drop in the RCR occurs very rarely, and this decrease may provide an accurate picture of the diminishing influence of that article relative to its peers [28]. Both the time of year when an article is published and the length of time that it is an online publication before its print publication date affect the RCR. For newer articles, the difference in time for citations to accrue between an article published in December versus one published in January can be quite significant.

Despite these issues, the RCR is arguably a significant improvement over the most commonly used metrics. There are still many open questions about the practical application of the RCR. For instance, there is no current agreement on how best to extrapolate from a set of RCRs (e.g., for all articles by an author) to a single number suitable for comparative purposes, like the h-index.

We have found that the administration at our institution has been receptive to the RCR as a supplement to or replacement for existing metrics. Our institutional bibliometrics dashboards [2] have been updated to replace displays of raw citation counts with a number of different visualizations incorporating the RCR. We have updated our library’s publication metrics tool to incorporate the RCR. The promotion and tenure reports currently provide the mean and median RCR values for a faculty member’s articles alongside the h-index.

As medical librarians devote more time and attention to bibliometrics, we are fortunate to have better tools at our disposal. We strongly urge medical librarians to add the RCR to the other arrows in their bibliometrics quiver.

## SUPPLEMENTAL FILES

Appendix A Research impact presentations at Medical Library Association annual meetings by year

Appendix B Top 2016 NYU School of Medicine publications

Appendix C NYU School of Medicine 2015 publications
