# Supplementary material for: The relative citation ratio: what is it and why should medical librarians care?
Source: J Med Libr Assoc. 2018 Oct 1;106(4):508–13. doi: 10.5195/jmla.2018.499 (PMC6148595; doi:10.5195/jmla.2018.499)
Supplement: Appendix A [file jmla-106-508-s001.pdf]

JMLA Journal of the Medical Library Association

Supplemental content to
J Med Libr Assoc. Oct;106(4):dx.doi.org/10.5195/jmla.2018.499
www.jmla.mlanet.org
© Surkis, Spore 2018

The relative citation ratio: what is it and why should medical librarians care?

Alisa Surkis, PhD, MLS; Stuart Spore, MS, MLIS, MS, MLIS

APPENDIX A

Research impact presentations at Medical Library Association annual meetings by year

Papers and posters found searching Medical Library Association (MLA) annual meeting programs using the search terms: metric, citation, impact.

MLA '17 (13)

Papers (note full panel on "Bibliometrics in Action")

1. Daring to Move Beyond the H-Index: Communicating Researcher Impact with the Relative Citation Ratio
2. Developing a Model for Faculty Scholarly Metrics Services across Diverse Health Sciences Audiences: From Dreaming to Doing
3. From Dreaming to Doing: Implementing Research Assessment Services (full session with 11 presenters from 7 libraries)
4. Keeping up with Demand: Doing National Institutes of Health Biosketch Support
5. HAMMERing out the Details: What Can an Online Bibliometrics Engine Tell Us about Research in Animal-Assisted Therapy
6. Implementing the Vision: Making Research Evaluation Services a Reality

Posters (note that there are tracks for posters starting in 2017, and one is "Metrics")

1. Assessing Research Impact: How Good Is Good Enough?
2. Daring to Think Outside the Box: Using Endnote to Create a Research Matrix
3. The Faculty Publications Metrics Project (FPM) at the New York University (NYU) Health Sciences Library
4. Library Outreach to Administrative Professionals in a Biomedical Research Institution
5. Novel Approach to Institutional Publication Tracking
6. Searching by Grant Number: Analysis of Web of Science and PubMed Search Results
7. Tracking Departmental Scholarly Publications

MLA '16 (9)

Papers

1. Mining for Gems: Gathering Research Impact Metrics with a DIY Approach
2. Research Impact and Evaluation Services in the Library: One Piece at a Time

Posters

1. Bibliometric Analysis and Funding Success to Evaluate an Organization's Research Grant Decisions
2. Continuing Altmetric Analysis
3. Evaluative Bibliometrics Meets the Clinical and Translational Science Institute
4. The Metric Mosaic: Evaluation of Research Impact
5. The Mosaic of Translation: An Analysis of Translational Medicine Publications
6. Publication Metrics: Services and Support
7. Refining an Automated Process for Tracking Institutional Publications

JMLA Journal of the Medical Library Association

SC

Supplemental content to
J Med Libr Assoc. Oct;106(4):dx.doi.org/10.5195/jmla.2018.499
www.jmla.mlanet.org
© Surkis, Spore 2018

MLA ’15 (8)

Papers

1. Design and Implementation of a Novel System to Quantify and Contextualize Research Impact
2. Pushing the Envelope: A Program for Illustrating Research Productivity and Impact Narratives

Posters

1. Further Altmetric Analysis
2. Impactful Visualizations of Bibliographic Metadata in Cardiovascular Disease Epidemiology
3. Library Helps Benchmark Research Performance of the Department
4. Pioneering a Research Impact Service
5. Using Altmetrics as Educational and Outreach Tools for Researchers: A Case Study

MLA ’14 (10)

Papers

1. Building a Better Translational Researcher: The Library’s Role in Training the Clinical and Translational Researcher
2. What’s the Difference between Research Metrics

Posters

1. Altmetrics Analysis
2. An Emerging Medical Library’s Endeavor to Track Institutional Scholarly Publications
3. Empowering Researchers to Increase Their Impact via Altmetrics and Open Access
4. Enjoying the Skyline: Providing Researchers with a View of Impact
5. Measuring Nursing Faculty Impact: Web of Science versus Scopus
6. New Measures of Success: Altmetrics and the Changing Face of Scholarly Impact
7. Use of Citation Metrics to Demonstrate Impact of Interdisciplinary Research Center
8. Web of Science versus Scopus for Capturing Researcher Output: A Bibliographic Comparison

MLA ’13 (4)

Papers

1. Altmetrics: Determining the Full Impact of Scholarship
2. Telling the Research Story: A Role for Librarians in Analyzing Research Impact Based on Evidence

Posters

1. Impact Factors: The Next Generation
2. Tracking National Library of Medicine Funding in Published Articles
